# In-ear infrasonic hemodynography with a digital health device for cardiovascular monitoring using the human audiome

**DOI:** 10.1038/s41746-022-00725-3

**Published:** 2022-12-22

**Authors:** Francis Roosevelt Gilliam, Robert Ciesielski, Karlen Shahinyan, Pratistha Shakya, John Cunsolo, Jal Mahendra Panchal, Bartlomiej Król-Józaga, Monika Król, Olivia Kierul, Charles Bridges, Christine Shen, Carly E. Waldman, Martin Ring, Tomasz Szepieniec, Anna Barnacka, Sanjeev P. Bhavnani

**Affiliations:** 1 Prisma Health USC Medical Group, 115 North Sumter Street, Suite 410, Sumter, 29150 SC USA; 2 MindMics, Inc., 86 Sherman Street, Cambridge, 02140 MA USA; 3 Healthcare Innovation & Practice Transformation Laboratory, Division of Cardiology, Scripps Clinic and Research Institute, La Jolla, 92037 CA USA

**Keywords:** Diagnostic markers, Data processing, Statistical methods

## Abstract

Human bodily mechanisms and functions produce low-frequency vibrations. Our ability to perceive these vibrations is limited by our range of hearing. However, in-ear infrasonic hemodynography (IH) can measure low-frequency vibrations (<20 Hz) created by vital organs as an acoustic waveform. This is captured using a technology that can be embedded into wearable devices such as in-ear headphones. IH can acquire sound signals that travel within arteries, fluids, bones, and muscles in proximity to the ear canal, allowing for measurements of an individual’s unique audiome. We describe the heart rate and heart rhythm results obtained in time-series analysis of the in-ear IH data taken simultaneously with ECG recordings in two dedicated clinical studies. We demonstrate a high correlation (*r* = 0.99) between IH and ECG acquired interbeat interval and heart rate measurements and show that IH can continuously monitor physiological changes in heart rate induced by various breathing exercises. We also show that IH can differentiate between atrial fibrillation and sinus rhythm with performance similar to ECG. The results represent a demonstration of IH capabilities to deliver accurate heart rate and heart rhythm measurements comparable to ECG, in a wearable form factor. The development of IH shows promise for monitoring acoustic imprints of the human body that will enable new real-time applications in cardiovascular health that are continuous and noninvasive.

## Introduction

Biometric monitoring is crucial to our understanding of health and disease. Despite our ability to generate increasingly large quantities of electronic health data, health care continues to be reactive, treating disease only after it is diagnosed. This approach narrows our capacity to implement preventative measures and assumes that every individual follows the common trajectories of disease and the course of treatments.

Physiological processes such as respiratory rate, heart rate, blood pressure, muscle activity, and internal movements of organs generate electric, thermal, chemical, and acoustic energy^1^. Biological signals representing aspects of these energies are transmitted as electric potential, pressure difference, mechanical vibrations, or acoustic waves, and can be measured using transducers and technologies attached to different parts of the body^1–6^. These digital health technologies (DHTs) include wearable and wireless devices^7–9^, smartphone-connected technologies^10,11^ implantable sensors^12,13^, and various lab-on-a-chip nanosensor platforms^14,15^. DHTs are constantly expanding and becoming increasingly sophisticated in their ability to quantify physiologic measurements through advanced computational approaches, thus challenging our contemporary methods for how physiologic parameters are measured, and ultimately how a disease is detected and monitored^10,16^.

DHTs are able to record a variety of signals, including cardiac activity, blood pressure, respiration, brain activity (EEG), and muscle activity (EMG)^9,11,17^. Cardiovascular measurements can be extracted from wearable DHTs, such as activity monitors and smartwatches using various methods including electrocardiography (ECG), photoplethysmography (PPG), oscillometry (pulse rate variation), biochemical sensors, or a combination of techniques^18^. These cardiac monitoring devices are commonly validated on standard physiologic measurements such as heart rate (HR), interbeat interval (IBI), and heart rate variability (HRV). While wearable devices are commercially available, most of them are not considered medical devices, as they are typically incomplete and inaccurate with an error rate of up to 10 percent in reporting HR alone^12,19^. Several other design constraints, including limitations in power consumption, memory, and data storage, impact the ability of consumer wearables for precise and continuous beat-to-beat measurements.

Herein, we introduce a method of in-ear infrasonic hemodynography (IH) and a technology platform that uses sensors embedded in earbuds to monitor cardiovascular activity. The IH technology aims to provide continuous and accurate cardiac signal measurements and to bridge the gap between convenient wearables and precise medical devices. Within the present investigation, we compare the IH performance to the gold-standard ECG using data from two dedicated clinical studies, with study subjects in sinus rhythm (SR, *n* = 25) and atrial fibrillation (AF, *n* = 15). First, we evaluate the accuracy of IH in measuring IBI and HR by correlations to ECG in healthy SR subjects and explore if this accuracy is preserved in the presence of large IBI variations induced by various breathing exercises. A similar correlation analysis is performed in AF subjects, for whom large IBI variations occur naturally, characteristic of the disease. Finally, we assess the IH ability to differentiate between AF and SR, by comparing it to ECG in a machine-learning approach for rhythm classification. In this approach, we build and train a random forest classifier model using additional external ECG data from patients in SR and AF (PhysioNet database). Then, without further modifications, the trained model is applied to our joint SR and AF data, separately for ECG and IH, and the IH performance in AF detection is compared to the ECG performance.

## Results

### Infrasonic hemodynography earbud system

The in-ear headphone with embedded IH technology shares a similar architecture to many consumer wireless in-ear headphones and can be used to collect IH signals and simultaneously play audio. Each earbud has an integrated acoustic sensor that enables the measurement of small fluctuations in in-ear acoustic pressure. The turbulence associated with the heart sounds and vascular hemodynamics has specific infrasound features that are captured by the IH earbuds^20^. While IH signals are captured below the range of human hearing (<20 Hz), audio output is conventionally restricted to within the range of human hearing (20 Hz to 20 kHz); thus, there is minimal interference in the IH signal when speaker audio is present. This phenomenon allows for new methods of physical acoustical tuning, which contribute to an earbud design that optimizes IH signal acquisition and at the same time preserves audio quality comparable to that of consumer-grade in-ear headphones.

**Fig. 1:**
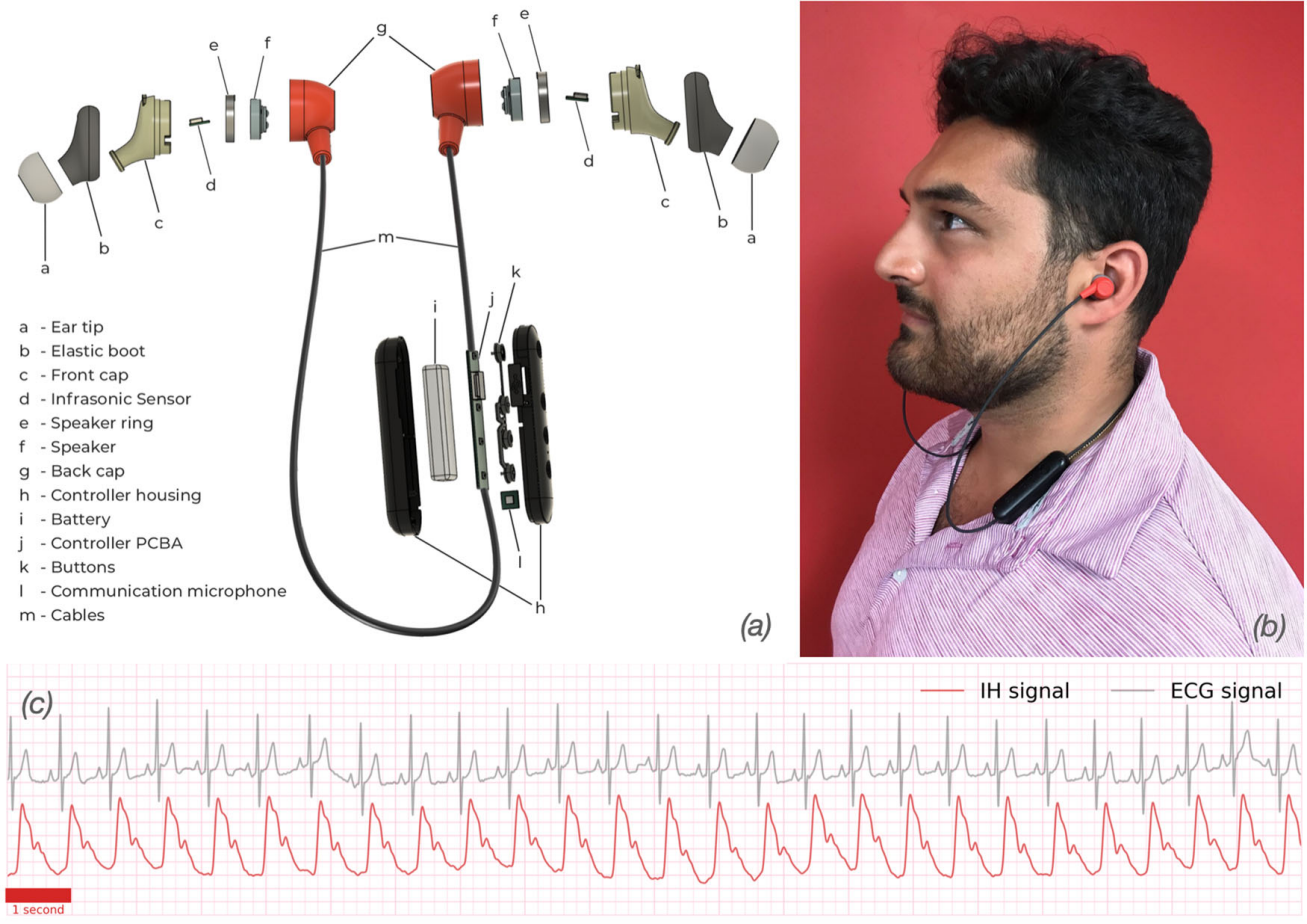
Infrasonic hemodynography earbud system. **a** Exploded schematic illustration of the IH earbuds, interconnect schemes, and enclosure architectures. **b** Person wearing the IH earbuds; the earbuds have passive sensors/microphones installed on the left and right side. **c** Illustrative example of IH signal collected alongside and synchronized with ECG. The person in the photograph wearing the earbuds consented to the taking and publication of the photograph.

A device layout and an image showing the in-ear headphone being worn are illustrated in Fig. 1a, b, respectively. The headphone consists of two earbuds and a controller that can communicate with mobile devices via Bluetooth Low Energy (BLE). The earbuds and the controller are connected via a cable (Fig. 1a–m). Each earbud contains the infrasonic sensor (Fig. 1a–d) and the speaker (Fig. 1a–f). The controller board (Fig. 1a–j) is placed in a controller housing (Fig. 1a–h), which in addition hosts the device battery (Fig. 1a–i) The infrasonic sensors are passive sensors that do not use a transmitter to send a signal, thus, the additional electric power required to operate them is minimal.

In the original design, the continuous data stream acquired using IH headphones is received via BLE on a mobile device. The device formats raw data and sends it using a secured communication protocol (Message Queue Telemetry Transport, MQTT) to a cloud infrastructure, where the data can be stored and processed in large quantities beyond the device memory. Such a design allows for continuous signal collection and processing without compromising data quality and sampling rate. For clinical studies presented here, to simultaneously record the data from IH and ECG, the wireless mobile device was replaced by a laptop computer connected to the controller through a USB cable (Fig. 7). The computer subsequently sent the joint data to the cloud. Figure 1c shows the exemplary signal collected from the in-ear headphone with embedded IH technology alongside signal from ECG for reference. IH and ECG signals were synchronized at the hardware level such that both ECG and IH used a common system clock to sample signals simultaneously.

### Infrasonic hemodynography signals

**Fig. 2:**
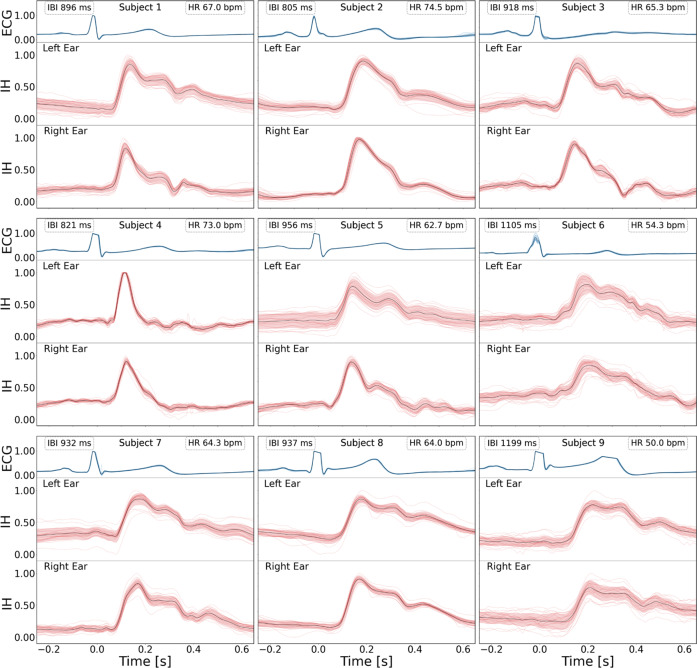
Exemplary signals from in-ear IH headphones and synchronized Lead-II ECG representing one cardiac cycle. ECG (top, blue) and IH signals from the left (red, middle) and right (red, bottom) ears for nine healthy subjects in the SR sample shown for 20 heartbeats stacked together (dotted lines), along with their mean (solid line) and standard deviation (band). Subjects' heart rate (HR) and the average duration of the cardiac cycle (IBI) are displayed as well.

Figure 2 presents examples of cardiac activity recorded in the left and right IH earbuds and ECG among study subjects in sinus rhythm (*n* = 9) when seated and breathing normally. For each subject, the data corresponds to 20 cardiac cycles, identified using ECG R-peaks reconstructed with a standard peak detection algorithm. Normalized signal waveforms of 20 individual heart beats were superimposed and shown together with their average and standard deviation, separately for IH and ECG. The distributions were centered at the position of ECG peak and shown for a time interval corresponding to 250 ms before and 650 ms after the ECG peak, independent of subjects’ heart rate. For a given study subject and a given channel (left or right), the variation between individual heart beats, expressed in terms of signal fidelity, ranged between 0.94 and 1.00, with a median of 0.97. The variation between left and right channels was larger (reflected by lower values of signal fidelity; between 0.88 and 0.99, with a median of 0.94), attributed to an expected difference in frequency response associated with the earbud placement in the ear canals. IH waveforms exhibit a prominent peak that is delayed with respect to the QRS complex of the ECG. The average delay between the ECG R-peak and the onset and the position of the IH peak was 84 and 158 ms for the left channel and 83 and 158 ms for the right-channel, respectively, showing good synchronization between the channels.

### Interbeat interval and heart rate measurements in sinus rhythm

**Fig. 3:**
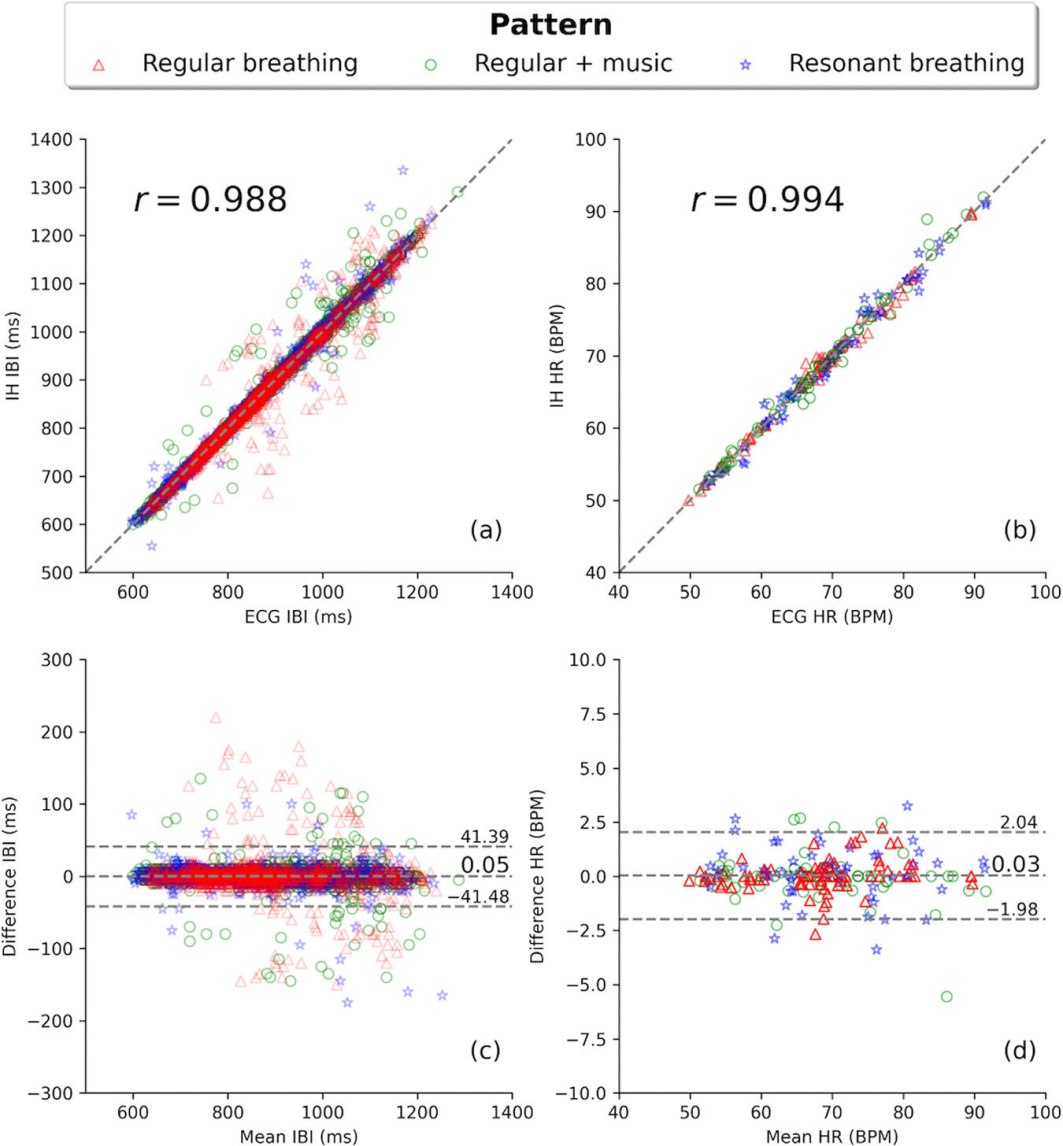
IBI and HR correlation analysis and the relationship of agreement between IH and Lead-II ECG. Correlation between **a** IBI and **b** HR, quantified by the Pearson correlation coefficient, *r*, and (**c**, **d**) the relationship of the agreement for simultaneous IH and ECG measurements. Separate markers correspond to three different breathing and environmental conditions, with subjects: (red triangle) breathing normally,(green circle) breathing normally while listening to music, and (blue star) performing resonant breathing. The identity line (IH IBI = ECG IBI) in top plots is drawn to guide the eye. The horizontal lines in bottom plots depict the mean value of the difference and the 95% confidence interval region: *d* and *d* ± 1.96*σ*, respectively.

The comparison between simultaneous IH and ECG measurements among study subjects in SR (*n* = 25) was based on 3744 IBI pairs and 198 HR pairs, with the latter calculated by averaging IBI within 20-second time windows. The correlation between the IH and ECG data (Fig. 3a, b) was *r* = 0.988 and *r* = 0.994 for IBI and HR, respectively. Figure 3c, d illustrates the relationship of agreement between IH and ECG measurements. The mean value of the difference was *d* = 0.05 ms and *d* = 0.03 bpm for IBI and HR, respectively, and a standard deviation of d was *σ* = 21.1 ms for IBI and *σ* = 1 bpm for HR.

Table 1 summarizes the correlation coefficient and the relationship of agreement between IH and ECG for the entire sample presented in Fig. 3, and for three separate subsamples, when subjects performed regular breathing, regular breathing while listening to music, and resonant breathing (also distinguished in Fig. 3 by separate colors). The consistency of results (*d* ± *σ*) for the three cases was investigated using the one-way ANOVA test (Table 2), which gave the F value equal to 1.8 and 0.8 for IBI and HR, respectively. The corresponding p-values were 0.16 for IBI and 0.44 for HR. This indicates that there is no significant difference in IH ability to measure IBI during rapid changes induced by resonant breathing or with external noise such as music.

**Table 1:** IBI and HR correlation analysis and the relationship of agreement between IH and ECG.

	No. of pairs	*r*	*d* ± *σ*
IBI			(ms)
All	3744	0.988	0.05 ± 21.1
Regular breathing	1448	0.979	0.8 ± 26.3
Regular + music	1176	0.992	− 0.6 ± 18.6
Resonant breathing	1120	0.994	− 0.5 ± 15.3
HR			(bpm)
All	198	0.994	0.03 ± 1.00
Regular breathing	80	0.997	0.03 ± 0.75
Regular + music	61	0.991	− 0.09 ± 1.12
Resonant breathing	57	0.994	0.15 ± 1.22

The number of IH and ECG pairs, the Pearson’s correlation coefficient (*r*), and the relationship of agreements (*d* ± *σ*) in the IBI and HR measurements for the entire SR sample and for separate subsamples with subjects performing regular breathing, regular breathing while listening to music, and resonant breathing.

**Table 2:** One-way ANOVA test.

	Sum of squares	d.o.f.	Variance	*F*	*p*
IBI					
Between groups	1827.0	2	813.5	1.82	0.16
Within groups	1669325.1	3741	446.2		
Total	1670952.2	3743			
HR					
Between groups	1.70	2	0.85	0.82	0.44
Within groups	203.05	195	1.04		
Total	204.75	197			

Results of a one-way analysis of variance (ANOVA) for the IBI and HR means from Table 1 (*d* ± *σ*) for three groups of subjects performing regular breathing, regular breathing while listening to music, and resonant breathing. Columns show the values of sum of squares (between and within groups, as well as the total sum), the number of degrees of freedom (d.o.f.), the variance between and within groups, the F statistics, and the corresponding *p* value.

### Sensitivity to physiologic changes

**Fig. 4:**
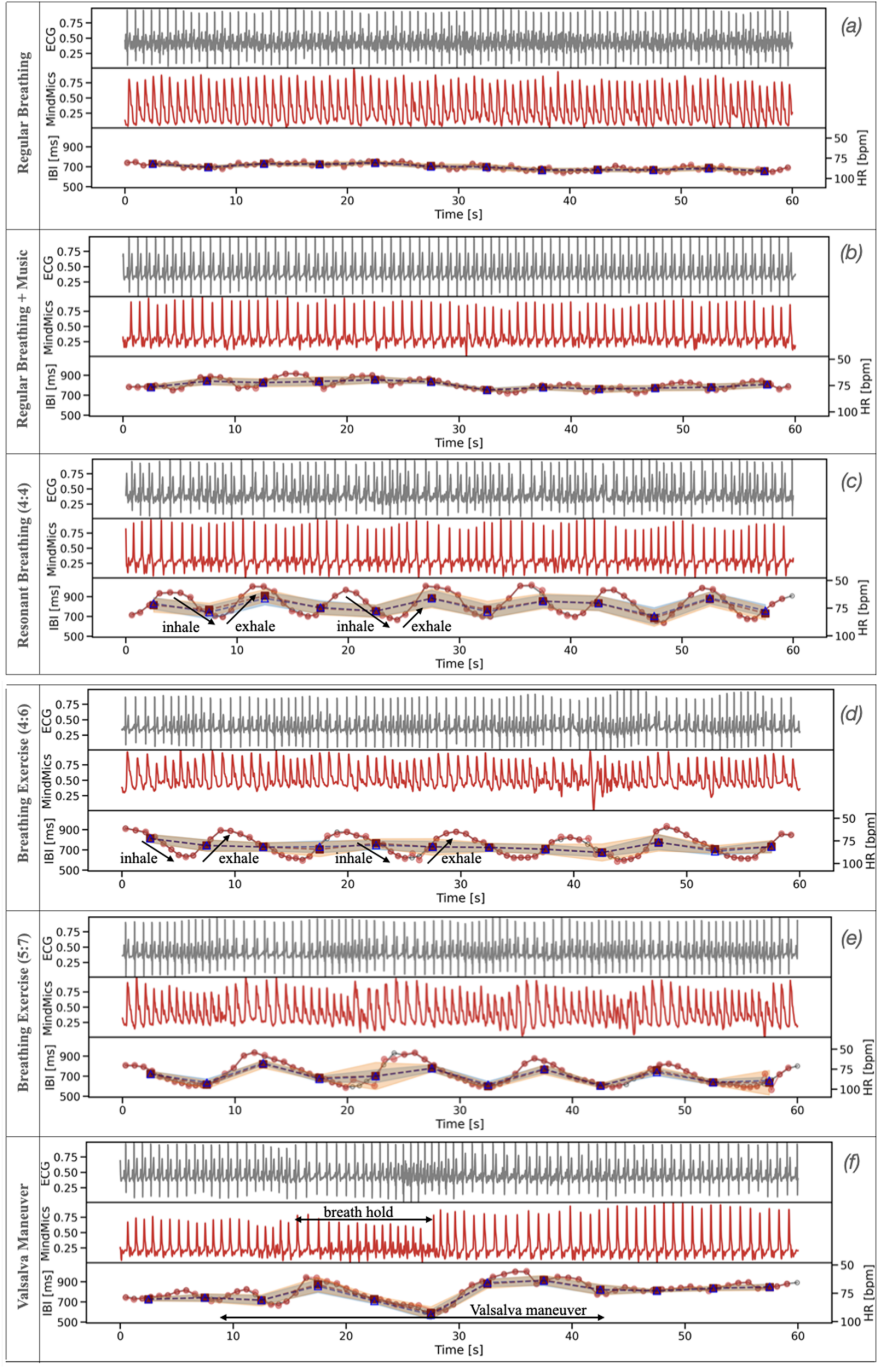
Physiological changes during minute-long breathing exercises. Comparison between various metrics calculated from ECG and IH signals during **a** regular breathing, **b** regular breathing while listening to music, and resonant breathing with the **c** 4:4, **d** 4:6, **e** 5:7 inhale-to-exhale ratio, as well as **f** the Valsalva maneuver. In each sub-figure, the top panel shows the ECG data (gray), the middle panel presents the IH data (red), while the bottom panel shows the IBI tachograms (left vertical axis) and HR and HRV values calculated from IBIs and averaged over 5 seconds (right vertical axis). The IBIs, HR, and HRV from the IH data are depicted as red circles, red squares, and orange bands, respectively. The same metrics from ECG data are shown as gray open circles, navy open triangles, and blue bands, respectively, although they are largely obscured by the IH data, hence hard to distinguish.

The time dependence of IBI patterns measured with IH and ECG for the three above-mentioned breathing exercises is illustrated in Fig. 4a–c. The regular breathing (with and without music) and the resonant breathing (with a 4:4 second inhale-to-exhale ratio) were performed by one of the subjects from the SR sample over a minute-long time interval. Figure 4d–f depicts examples of additional three breathing maneuvers that maximize IBI variations, namely, the resonant breathing with the 4:6 and 5:7 ratios, and the Valsalva maneuver. Low variability was present during regular breathing (Fig. 4a). The average HR measured from both IH and ECG was 84.7 bpm. The HRV was 22 ms from IH and 17 ms from ECG. Both HR and HRV were relatively constant during data collection. In contrast, during the resonant breathing, a change in IBI was 300 ms with an average HR change of 7 bpm, for all three inhale-to-exhale ratios (Fig. 4c–e). For these highly periodic breathing patterns, the respiratory rate can be measured using power spectral density, as discussed in Supplementary Note 1 and Supplementary Fig. 1. Finally, during the Valsalva maneuver, IBI decreased by over 300 ms and rebounded by ~400 ms at the end of the maneuver (Fig. 4f). The amplitude of the IH signal followed a similar pattern as the IBI, showing a monotonous drop followed by a sudden increase.

The level of agreement between IH and ECG in tracking time-dependent IBI variations from Fig. 4 was investigated using a two-sided paired *t* test. Table 3 presents the resulting values of test statistics and the corresponding *p* values, which indicate that IH-tracked IBI variations were similar to ECG.

**Table 3:** Agreement between IH and ECG in time-dependent IBI variations.

	Regular	Regular	Resonant	Resonant	Resonant	Valsalva
		+ Music	(4:4)	(4:6)	(5:7)	
Paired *t* test:	0.087	0.188	0.118	−0.057	0.127	−0.537
	(0.93)	(0.85)	(0.91)	(0.95)	(0.90)	(0.59)

Test statistics and *p* values (in brackets) computed from the two-sided paired *t* test for six different breathing exercises performed over a minute-long time interval (d.o.f. = 72–86) by one of the subjects in the SR sample.

### Interbeat intervals in atrial fibrillation

**Fig. 5:**
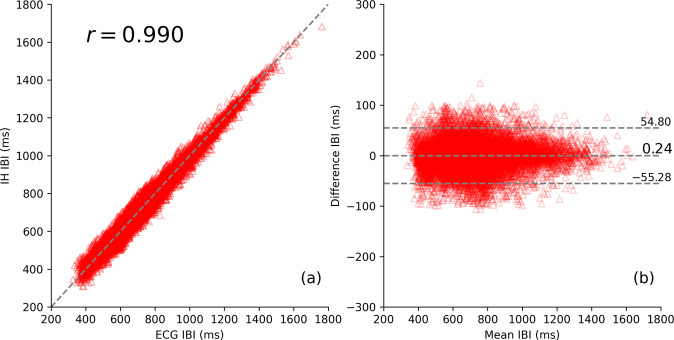
IBI correlation analysis and the relationship of agreement between IH and ECG in the AF sample. **a** Correlation between IBI and **b** the relationship of agreement for simultaneous IH and ECG measurements in the AF sample. The identity line in the left plot is drawn to guide the eye. The horizontal lines in the right plot depict the mean value of the difference and the 95% confidence interval region: *d* and *d* ± 1.96*σ*, respectively.

The ability to accurately measure IBI with IH was also explored using data from patients with AF. In contrast to regular SR patients, for whom large IBI variations at a short time scale can be induced by breathing exercises, the AF condition allows us to access even larger and irregularly changing IBI values, occurring naturally due to patients’ intrinsic heart condition. Figure 5 shows the IBI correlation between IH and ECG for subjects in the AF Study (*n* = 15). As expected, the AF data span a broader range of IBI values (400–1600 ms) than the SR data (600–1200 ms, Fig. 3). The IH and ECG correlation in the extended IBI region remained high (*r* = 0.99).

### Distinguishing atrial fibrillation from sinus rhythm

The comparison of IH to ECG was further investigated by quantifying their ability to detect AF. The AF detection was performed by means of a machine-learning algorithm based on a random forest classifier, trained and tested using >200,000 30-second segments of external publicly available ECG data with AF and SR rhythms from PhysioNet database (11,054 and 196,514 segments, respectively). Table 4 presents the results of the model performance when applied to classify AF and SR rhythms in individual IH and ECG data from the combined AF and SR samples from this study. The confusion matrices for distinguishing AF from SR are presented in Tables 5 and 6 for the ECG and IH samples, respectively. There were five and four AF segments misclassified as SR in the ECG and IH data, respectively. No SR segments were misclassified as AF in the ECG data, while 5 SR segments were misclassified as AF in the IH data. Overall, the algorithm demonstrated equally good performance (Sensitivity, Specificity ≥0.99) for distinguishing between AF and SR for both IH and ECG.

**Table 4:** Results of machine learning algorithm for detecting AF rhythms in the ECG and IH data.

	No. of subjects	No. of segments	Total time (s)	Accuracy	Precision	Recall
ECG	SR: *n* = 25	606	18,180	1.00	0.99	1.00 [0.99, 1.0]
	AF: *n* = 15	458	13,740		1.00	0.99 [0.98, 1.0]
IH				0.99	0.99	0.99 [0.98, 1.0]
					0.99	0.99 [0.98, 1.0]

Number of SR and AF subjects, number of 30-second segments of cardiac rhythms and their total duration (common for the ECG and IH data), as well as accuracy, precision, and recall metrics for detecting AF rhythms with a random forest classifier model. The model was trained and validated using external PhysioNet ECG data and then applied to individual ECG (top row) and IH data (bottom row). Recall for the AF and SR samples corresponds to Sensitivity and Specificity in detecting AF, respectively. Numbers in square brackets adjacent to Recall values correspond to confidence intervals (95%) calculated as Wilson score intervals^21^.

**Table 5:** Confusion matrix for ECG segment classification.

ECG	Predicted	SR	AF
Actual	SR	606	0
	AF	5	453

Number of predicted SR and AF segments (columns) shown separately for each actual (true) segment type (rows) in the ECG data.

**Table 6:** Confusion matrix for IH segment classification.

IH	Predicted	SR	AF
Actual	SR	601	5
	AF	4	454

Number of predicted SR and AF segments (columns) shown separately for each actual segment type (rows) in the IH data.

## Discussion

In the present work, we introduced an IH technology that uses infrasonic sensors embedded in earbuds for continuous monitoring of cardiovascular activity. We explored IH capabilities for precise beat-to-beat assessment by comparing its performance to the gold-standard ECG using data collected in SR and AF subjects. The results of our investigation can be summarized as follows:

- Waveforms of consecutive heartbeats in SR exhibit high signal fidelity (0.94–1.00) and show a prominent peak whose onset and position are delayed with respect to the ECG QRS complex by approximately 80 and 160 ms, respectively;
- A direct comparison of IBI and HR values measured from simultaneous IH and ECG signals in SR subjects shows a high correlation (*r* = 0.989 and 0.994 for IBI and HR, respectively);
- Tachograms with time-dependent beat-to-beat variations in IBI measured with the IH data demonstrate a similar variability (300–400 ms) among resonant breathing exercises and the Valsalva maneuver;
- IH demonstrates high correlation (0.99) with heart rate variation in AF and between simultaneous IH and ECG measurements;
- A random forest classifier trained to distinguish AF from SR using publicly available ECG data, shows high performance (Sensitivity, Specificity ≥0.99) when applied separately to the IH and ECG data of the same cardiac rhythms.

IH waveforms correlate with the cardiac cycle with high signal fidelity. Small deviations from the unity in fidelity values are due to differences between consecutive beats, which may occur due to physiological changes, the sensitivity of acoustic sensors, or minor changes in the placement of earbuds during wear. Differences in waveforms between the left and right channels are attributed to differences in earbuds placement between the channels. Notable differences between the subjects are predominantly physiological.

A comparison between IBI, HR, and HRV metrics calculated using IH and ECG signals during breathing exercises inducing physiological changes shows that in-ear headphones with embedded IH technology are capable of capturing IBI changes at short timescales making it possible to continuously monitor heart rate and heart rate variation. Our preliminary methods of monitoring autonomic nervous system response through IBI tachograms (breathing and Valsalva) can potentially be extended towards clinical applications where closed-loop biofeedback is measured and augmented, e.g., for sleep and stress monitoring.

The present study of cardiac rhythms focused specifically on SR and AF provides a basis for IH earbuds and for longer-term arrhythmia monitoring. The high sensitivity to detect AF during short time segments is especially critical for identifying and characterizing paroxysmal AF that may be particularly relevant in asymptomatic AF and for AF screening^21–26^. Future studies are underway to validate these findings in larger datasets and to include a variety of cardiac arrhythmias (Supplementary Fig. 2). Given the ubiquity of the earbud form factor^27^, it carries additional advantages, such as extended wear time and comfort, suitable for day time and night time monitoring.

A number of limitations need to be appreciated when interpreting our results on distinguishing AF from SR. First, given a small sample size, it is possible that our results may overestimate the accuracy of AF classification and the correlation statistics between IH and ECG. Second, only the sensitivity and specificity of the algorithm were evaluated. As the study sample increases in size and among cohorts with different prevalence of AF, correlation statistics such as positive and negative predictive values will be different. Third, the AF classification algorithm relies only on IBI and will not recognize AF if the cardiac rhythm appears regular despite other AF signatures, such as a missing P-wave. Fourth, the current study and the associated algorithms do not directly assess the ability of IH to detect other arrhythmias and successfully separate them from AF and SR, although other arrhythmias are present in the AF sample (Supplementary Fig. 2). The inclusion of other rhythms is expected to reduce the performance of the classification algorithm. A separate system will be required to apply IH for AF detection in the general population that can be trained and validated for the identification of other arrhythmias using the methods described here.

Our future work will also focus on applying the IH technology to provide insights into associated hemodynamics not accessible with ECG. While the ECG signal provides information about the electrical activity of the heart that triggers the heart contraction, IH monitors mechano-acoustic signals originating from the heart contraction and relaxation such as aortic valve opening and closing or pressure pulse waves that travel along the vascular system into the ear canal. The IH waveform exhibits similarities to the aortic pressure waveform in cardiac catheterization, and could potentially be used, e.g., to measure systolic and diastolic time intervals of the heart cycle. By developing algorithms to detect these and related cardiac functions, the IH technology may be expanded toward comprehensive monitoring of the cardiovascular system and the early detection of cardiac dysfunction in a noninvasive and continuous way.

The in-ear IH technology is based on passive detection of low-frequency acoustical biosignals associated with the vascular hemodynamics, amplified through a pressure increase in the sealed ear-canal cavity. Signals are detected by acoustic pressure sensors and propagated to the controller board for data acquisition. In this study, to simultaneously collect IH and ECG data, the controller PCBA was connected to the laptop computer through a cable. In the original design, the system uses BLE to communicate with the mobile device (primarily for an extended battery life even when data is being streamed continuously), which sends the data to the cloud infrastructure. Currently, the BLE-embedded version has cable connections between the electrical components of each earbud and the controller (Fig. 1a). In the future, for convenient wear and signal coverage increase, the IH technology will be implemented as truly wireless in-ear headphones. By using online servers in the cloud, the system is able to perform continuous, real-time data collections and analysis without the problems of battery usage, storage, and complex computations related to big data^28^. It also allows for instantaneous quality assessment, thereby enabling true closed-loop capabilities.

The results presented in this work are based on the data collected using a prototype device, with cables connecting the earbuds to the controller board. Additional cables were used to connect the controller board to the ECG device and the data-acquisition computer. This configuration made the system sensitive to additional vibrations propagating to the earbuds through the cables. Additional non-stationary signals, typically induced by subjects’ motion or environmental background, could have an amplitude significantly greater than cardiac signals or even saturate the system. The reported analysis aimed at demonstrating the potential of the IH technology and was based on periodic heart beats from subjects instructed to remain at rest. A complete analysis of motion signals (such as moving, walking, eating, etc.) is beyond the scope of this paper and will be addressed separately with fully wireless earbuds, additionally equipped with motion sensors.

The IH technology can be enabled in a wearable form factor in everyday earbuds, provided they ensure a proper occlusion. The technology uses microphones that are placed inside the earbud’s front cavity, which is the industry’s standard microphone placement, already present in earbuds with active noise cancellation (the feedback microphone). Moreover, the technology uses algorithms and data storage in the cloud, which saves the battery on the device with no compromise on accuracy. As a result, the system can collect the data for an extended period of time without any time gaps for power or memory preservation. The development of future technologies (e.g., 5G or quantum computing) and the reduction in costs will further propel the adoption of wearable devices by enabling faster data transfer rate, low end-to-end latency, reduced cost, increased computational power and improved device connectivity, opening path toward closed-loop applications and detecting diseases in presymptomatic people^29,30^.

## Methods

### In-ear infrasonic hemodynography

The in-ear headphone with embedded IH technology features a pair of audio earbuds that house acoustic and auxiliary sensors, which detect acoustic and mechanical vibrations from the ear canal. Acoustical biosignals from IH represent the fluctuating pressure changes inside the ear of an individual with respect to a reference pressure (e.g., ambient pressure). These biosignals typically contain sounds in the infrasonic range (0–20 Hz), which are sounds with frequencies below the audible range (20–20 kHz). Thus, infrasonic biosignals can be identified as acoustic signals with components in the “low-frequency” spectrum (i.e., from 0 to 20 Hz).

The acoustic pressure within a cavity, *P* (in Pascals), assuming constant temperature, is given by:

1 $$P={P}_{\rm{atm}}+{U}_{a}\cdot {Z}_{a},$$

where *P*_atm_ is static pressure (atmospheric pressure, 101.325 kPa at sea level), *U*_*a*_ is acoustic volume velocity (m^3^/s), and *Z*_*a*_ describes acoustic impedance of the cavity (*P*a⋅s/m^3^)^31^. Sound waves with an input acoustic volume velocity *U*_*a*_ interact with the acoustic impedance *Z*_*a*_ of the cavity. The product of these two variables is *P*_Δ_, the dynamic acoustic pressure of sound observed in the cavity. The complex acoustic impedance *Z*_*a*_ of a cavity with dimensions much smaller than the wavelengths of sound considered can be approximated as:

2 $${Z}_{a}\approx \frac{\rho {c}^{2}}{(j\omega )V},$$

where *ω* = angular frequency (Hz), *ρ* = the density of the fluid medium (1.21 kg/m^3^ in air), *c* = the speed of sound of the fluid medium (343 m/s in air), and *V* = the volume of the cavity (m^3^)^32^.

In the case of IH, both the biosignals generated by the human body and the audio emitted from the speaker of the earbud are observed as acoustic pressure changes in the ear canal. The occluded human ear canal has an average volume on the order of 2 cubic centimeters (*cc*). The smallest wavelength of sound within the infrasonic range (≤20 Hz) is 17.15 meters; therefore, the occluded ear canal is a suitable acoustic cavity for application of Equations (1) and (2) to the acoustic pressure of biosignals in the ear canal.

**Fig. 6:**
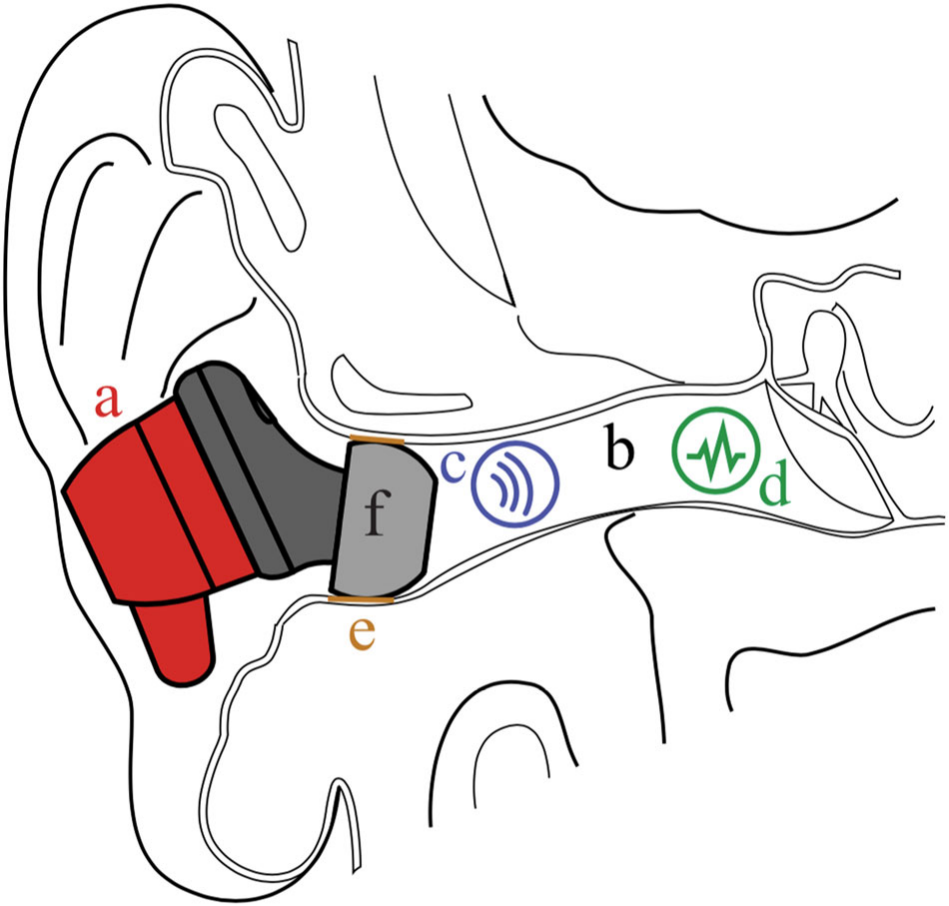
Placement of the earbud in the ear canal. The IH earbud (**a**), fitted into the ear canal (**b**). The earbud has capabilities to emit audio (**c**) and detect biosignals (**d**) within the infrasonic range. The seal (**e**) between the ear tip (**f**) and the ear canal wall plays a key role in the earbud’s capability of detecting biosignals.

Looking out of an open ear canal, which has a practically infinite volume, *Z*_*a*_ is negligible, and the acoustic pressure inside the ear canal becomes the atmospheric pressure. When an earbud is placed in such a way that the ear tip creates an airtight seal with the ear canal, thus creating a closed volume (Fig. 6), the acoustic impedance rises, and sound pressure fluctuations inside the ear canal are amplified, particularly within the infrasonic range.

The acoustic pressure and acoustic volume inside the ear canal are inversely related (*P* ⋅ *V* = const, Boyle’s ideal gas law). Occluding the ear canal significantly decreases its volume and accounts for a correspondingly large increase in dynamic acoustic pressure. For instance, shrinking the effective acoustic volume of the ear canal from 200 cc to 2 cc amplifies the acoustic pressure of biosignals by up to 40 dB. If the earbud can be fit in such a way that the effective acoustic volume of the ear canal shrinks by half, then the acoustic pressure magnitude of biosignals will subsequently increase by up to 6 dB. Such a significant boost to the amplitude of the acoustic signal brings human biosignals into measurable range with commercially available microphones. The seal also blocks external environmental noise, further improving biosignal detection.

### Datasets and collection protocols

#### SR sample

**Fig. 7:**
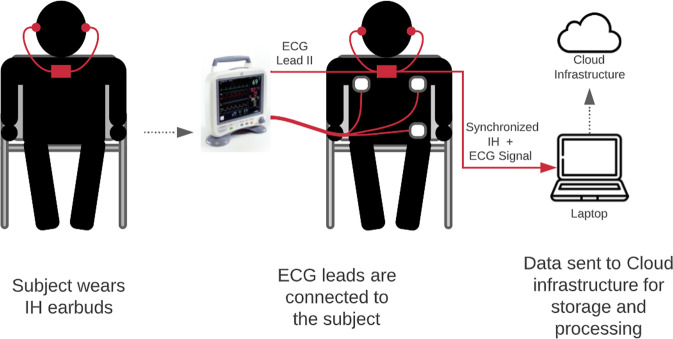
Clinical setup. Clinical setup for simultaneous data collection using ECG and IH. The drawing was created in Lucidchart, www.lucidchart.com.

The protocol was approved by the New England institutional review board (ClinicalTrials.gov Identifier: NCT05095753; start date: 15 November 2019, ongoing). Consecutive 25 healthy subjects (ages between 20 and 77 years, with a mean age of 42 years, and 35% female) were recruited at MindMics Inc. in Cambridge, MA for a prospective study of clinical validation of the in-ear headphones with embedded IH technology with simultaneously captured ECG waveforms. All study subjects provided written informed consent. As schematically depicted in Fig. 7, study subjects wore the IH earbuds in their left and right ears and were fitted with different-sized eartips to ensure a proper occlusion. A medical-grade 3 lead ECG (GE Transport Pro) was connected to each study subject’s left chest, right chest, and left leg to obtain reference signals. A time-series dataset was acquired through synchronized IH and ECG. Data for both IH and ECG devices were recorded with a sampling frequency of 1000 Hz. Data collection started with subjects seated upright and breathing normally. Subjects were then asked to perform a series of breathing maneuvers and introduced to soothing music to intentionally change respiratory rate and HR for a larger subset of data.

Analog Lead-II signal data from the ECG machine was sent to the IH earbuds where it was time-synchronized with the IH signal from the left and right ear. The signals were collected using a data acquisition device (DAQ) and sent to a laptop computer over a wired USB connection. The computer encrypted the signal and securely sent it to the cloud infrastructure for storage and further processing.

#### AF sample

The prospective study recruiting AF patients was approved by the institutional review board of The University of South Carolina School of Medicine (ClinicalTrials.gov Identifier: NCT05103579; start date: March 24, 2020, end date: 31 January 2021). The objective of this study was to evaluate the efficacy of the IH waveforms to monitor cardiac activity from participants with known atrial fibrillation. Consecutive study subjects were enrolled at Prisma Health in Sumter, SC, and underwent simultaneous IH and ECG recording for 20 minutes. Each study subject provided written informed consent. Study subjects with a known history of AF (*n* = 17) were screened and those in a rhythm of atrial fibrillation were included for participation (*n* = 15, ages between 45 and 90 years, the mean age of 71 years, and 47% women). The data collection process is schematically described in Fig. 7, with a clinical setup identical to the one used for the SR sample.

#### Study population

Table 7 shows an overall demographics for the subjects in the SR (*n* = 25) and AF (*n* = 15) samples. The detailed demographic data of SR subjects are presented in Supplementary Table 1, while the demographic data and comorbidities of the AF subjects are shown in Supplementary Table 2.

**Table 7:** Overall study demographic.

	SR sample	AF sample
Number of patients	25	15
Age range (average)	20–75 (42)	45–90 (71)
Sex (F/M)	9/16	7/8
Cardiovascular disease	-	Atrial fibrillation (11)
		Permanent atrial fibrillation (3)
		Atrial fibrillation with atrial flutter (1)
Comorbidities	-	Hypertension (8)
		Hyperlipidemia (5)
		Chronic obstructive pulmonary disease (3)
		Cardiomyopathy (2)
		Coronary artery disease (1)
		Aortic stenosis (1)
		Mechanical aortic valve replacement (1)
		Aortic valve regurgitation (1)
		Mitral valve regurgitation (1)
		Tricuspid valve regurgitation (1)
		History of aortic aneurysm dissection (1)
		History of ablation for atrial fibrillation (1)
		Thoracic aneurysms (1)
		Gastroesophageal reflux disease (1)

General clinical characteristics of subjects in the SR and AR samples. Numbers in brackets next to cardiovascular conditions correspond to the total number of patients diagnosed with those conditions.

For the study of agreement between IH and ECG measurements of IBI pairs using Bland-Altman plots, the minimum sample size (type I error = 0.05, type II error = 0.1) was estimated. Based on our preliminary measurements, an expected mean of differences of 1 ms, expected standard deviation of differences of 25 ms, and a maximum allowable difference of 55 ms were used for the calculation, resulting in a minimum sample size of 785 ^33,34^.

### Physiologic maneuvers

To induce and examine large variations of IBI patterns in the SR rhythm the following breathing maneuvers were suggested to the subjects:

- Regular breathing: a pattern that corresponds to the baseline changes in physiology and low levels of variability.
- Resonant breathing: a breathing exercise with a specific inhale-to-exhale ratio that induces large amplitude sinusoidal IBI patterns, known as respiratory sinus arrhythmia (RSA)^35^.
- The Valsalva maneuver: a way to transiently increase intrathoracic pressures, commonly performed by moderately forceful exhalation against a closed airway. This method leads to dramatic changes in the systemic blood pressure and HR that the autonomic nervous system attempts to compensate for and correct^36^. Here, the subject performed the bearing down method to induce the Valsalva maneuver.

These breathing techniques induce the response of the autonomic nervous system^37^. The changes in tachograms over the span of a breathing cycle may reflect the balance between sympathetic and parasympathetic nervous systems. RSA and changes in HRV related to stress are indicators of this balance^38^.

### Signal processing pipeline

**Fig. 8:**
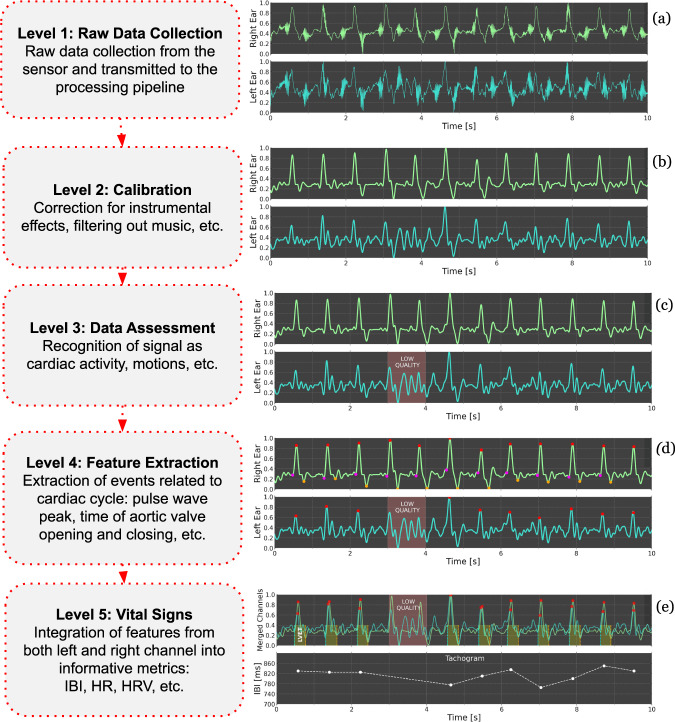
Schematic structure of data processing pipeline. The data processing pipeline which is used to compute online vital signs starting from raw signals collected by the earbud sensors. Each Processing Level (from 1 to 5) is characterized (left) and illustrated with example data (right) in a separate row of the figure.

Figure 8 introduces a conceptual structure of the processing pipeline optimized for sinus rhythm data, with general characteristics of each of the five data processing levels (Level 1–5). The pipeline performs on a live stream of data and results are delivered in a few seconds after data collection.

- Level 1: Raw data is collected from right and left earbuds. At this stage, the data may contain signals or artifacts caused by head movements, steps, music, etc., not originating from the cardiovascular system. Figure 8a shows an example of a biosignal obtained while listening to loud music (maximum volume) with strong bass.
- Level 2: Calibration occurs with corrections made for audio playing through the earbud and filtering of audible frequency range from speakers. Figure 8b illustrates that even in extreme cases music can be successfully filtered out from biosignals.
- Level 3: The data quality assessment is performed. Raw signals of a duration of one second are classified as either good-quality cardiac signals or loud signals generated by user motion using a proprietary neural network-based algorithm. The algorithm is based on a multi-layer perceptron classifier, which uses >20 features extracted from the signal in the time domain as an input. These features include statistical variables, such as a mean value and the variance of the signal strength, quantiles and the skewness and the signal distribution, etc., as well as morphological features, such as a signal shape, the signal peak width, and height, etc. The classifier was trained based on a machine-learning approach using manually classified data from about 70 subjects collected in a dedicated study, supported by simultaneous ECG signals. In total, >10,000 cardiac cycles were included in the model training, with ~50% of good-quality signals and 50% of signals with motion artifacts. For further analysis, only good-quality cardiac signals are selected, based on the value of a classifier output probability for a given signal window. An example of a low-quality signal due to motion artifacts rejected by this system is shown as a shaded area in Fig. 8c.
- Level 4: Events related to the cardiac cycle are extracted. Peaks in the biosignal waveforms are detected using an adaptive threshold method. Additional features are identified from the waveforms, including features that may correspond to cardiac events, such as aortic valve opening and closing (Fig. 8d).
- Level 5: Values of vital signs are calculated using features identified in Level 4. IBIs are obtained by measuring time intervals between consecutive peaks (Fig. 8e, bottom). Information from both earbuds is merged for improved reliability, favoring the channel with a higher score in the data assessment from Level 3. The joint signal is the basis for obtaining HR and HRV for a specific time window. Additional metrics, like respiratory rate, can be computed by combining both the sequence of peaks and features obtained from raw signals.

### Statistical analysis

#### Waveform fidelity

IH waveform fidelity is quantified as the median value of cross-correlation function maxima for successive waveform pairs. For each successive pair of waveforms, the cross-correlation function is calculated as the time-dependent Pearson correlation coefficient (*r*_*xy*_) for a range of lag times, *τ* between 0 and 500 ms:

3 $${r}_{xy}(\tau )=\frac{\mathop{\sum }\limits_{i=1}^{n}({x}_{i}-{x}_{m})({y}_{i+\tau }-{y}_{m})}{\sqrt{\mathop{\sum }\limits_{i=1}^{n}{({x}_{i}-{x}_{m})}^{2}\mathop{\sum }\limits_{i=1}^{n}{({y}_{i}-{y}_{m})}^{2}}},$$

where *x* and *y* are successive waveforms discretized into *n* 1-ms samples, and their mean values are given by *m*_*x*_ and *m*_*y*_, respectively. Waveform fidelity is then defined as *F* = median(max(*r*_*xy*_)). For the waveform comparison between the left and right channels, the *x* and *y* variables in the formula in Equation (3) are replaced by the left- and right-channel data of the same heartbeat.

#### Machine-learning model for AF detection

The current state-of-art AF detection algorithms are predominantly based on deep learning approaches that perform automatic feature extraction from input ECG waveforms and cardiac rhythm classification^39^. For our purpose, to compare cardiac rhythm classification with significantly different ECG and IH waveforms, a traditional machine learning algorithm was employed, with input features derived from IBI tachograms. A random forest classifier was first trained on publicly available ECG datasets with AF and SR rhythms and then used to classify AF and SR segments in individual IH and ECG datasets from combined AF and SR samples.

The random forest model was trained using external ECG databases from PhysioNet^40^, namely MIT-BIH Atrial Fibrillation, MIT-BIH Normal Sinus Rhythm, and Normal Sinus Rhythm RR Interval datasets. The algorithm relied entirely on interbeat intervals present in tachograms of the duration of 30 seconds. R-peaks were detected with an automatic algorithm and manually reviewed. Samples where the peak detection failed or which contained <10 IBIs were rejected. In total, 196,514 30-second SR segments (*n* = 72 patients) and 11,054 AF segments (*n* = 25 patients) were used with an 80–20 train-test split to train and internally test the classifier. 17 features that trace dispersion, degree of randomness, and frequency characteristics of tachograms were calculated and used as inputs to the model. The average number of samples per feature was about 10,000, sufficient for reliable model training^41^. Hyperparameter tuning was performed using five-fold cross-validation on the training set. Model parameters that maximized the precision metrics for the AF sample were used to train the model on the entire training set. The trained algorithm had accuracy = 0.98, sensitivity = 0.990 [0.987, 0.991], and specificity = 0.978 [0.978, 0.979], evaluated using the testing set. The numbers in brackets correspond to Wilson score intervals (95% CI)^21^.

**Fig. 9:**
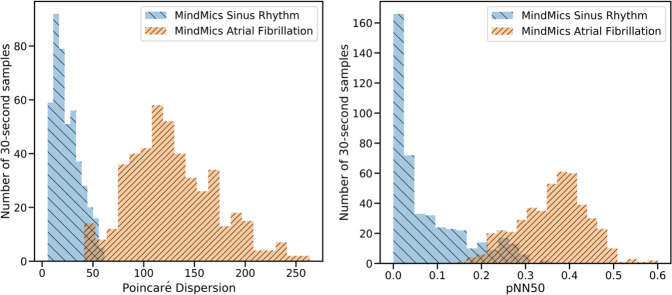
Examples of features used for AF and SR classification. Distributions of (left) Poincaré dispersion and (right) pNN50, the fraction of successive IBI >50 ms measurements, for 30-second SR and AF samples from the IH data. IBI interbeat interval, SR sinus rhythm, AF atrial fibrillation.

The model was then applied to the data of the current studies. All ECG data collected from subjects in the AF sample was adjudicated by a cardiac electrophysiologist for cardiac rhythm identification, and subjects with confirmed AF (*n* = 15) were selected. The dataset was complemented by the subjects from the SR Study (*n* = 25). As our signal processing pipeline described above was optimized for regular sinus rhythm, a dedicated AF sample selection was performed using the following criteria. For both IH and ECG data, quality assessment rules were applied to exclude data containing artifacts due to motion or from an improper fit of the earbuds. The data quality assessment was further visually verified and adjusted by members of the analysis team. An automated peak detection algorithm was applied to the IH and ECG data, and IBI were calculated between successive peak positions. In order to exclude rare heartbeats for which there was a clear electrical signal but limited blood flow and, hence, limited IH signal, an outlier rejection was performed on the resulting IBI, excluding IBIs falling four standard deviations away from the mean (∣IBI_IH_ − IBI_ECG_∣ < 100 ms) or outside of the 300–2500 ms range. The same procedures were applied to the SR sample used in this study. Data were divided into 30-second segments, requiring that a segment contained at least 10 IBI, resulting in 606 SR and 458 AF segments. These segments were then used to calculate the same set of 17 features, which were input to the machine learning algorithm described above, run separately on the IH and ECG data. Figure 9 shows the distribution of two features used in the algorithm that provided the highest separability of AF and SR samples (IH data).

### Reporting summary

Further information on research design is available in the Nature Research Reporting Summary linked to this article.

## Supplementary information

Supplementary Material

written consent, human subject picture

Clinical Trial information - 1

Clinical Trial information - 2 (SR)

Clinical Trial Information - 3 (AF)

Reporting Summary checklist

## Data Availability

Datasets used for the analyses in this study are available from the corresponding author upon request.

## Code availability

Models and codes generated or used during the study are proprietary or confidential in nature and may only be provided with restriction.
