# Supplementary material for: In-ear infrasonic hemodynography with a digital health device for cardiovascular monitoring using the human audiome
Source: NPJ Digit Med. 2022 Dec 22;5:189. doi: 10.1038/s41746-022-00725-3 (PMC9780339; doi:10.1038/s41746-022-00725-3)
Supplement: Supplementary file 1 — Supplementary Material [file 41746_2022_725_MOESM1_ESM.pdf]

Supplementary Material to

In-ear Infrasonic Hemodynography with a Digital Health Device for Cardiovascular Monitoring Using the Human Audiome

Francis Roosevelt Gilliam III[b], Robert Ciesielski[a], Karlen Shahinyan[a], Pratistha Shakya[a], John Cunsolo[a], Jal Mahendra Panchal[a], Bartłomiej Król-Józaga[a], Monika Król[a], Olivia Kierul[a], Charles Bridges[a], Christine Shen[c], Carly E. Waldman[c], Martin Ring[a], Tomasz Szepieniec[a], Anna Barnacka[a], Sanjeev P. Bhavnani[c]

From [a]MindMics, Inc, Cambridge, MA, [b]Prisma Health USC Medical Group, Sumter, SC, and [c]Healthcare Innovation & Practice Transformation Laboratory, Division of Cardiology - Scripps Clinic and Research Institute

Supplementary Note 1: Power spectra 2
Supplementary Figure 1: Power spectra of inter-beat interval tachograms 2
Supplementary Figure 2: Cardiac Rhythms observed in subjects from AF study 3
Supplementary Table 1: Characteristics of recruited healthy SR patients 4
Supplementary Table 2: Characteristics of recruited AF patients. 5
Supplementary References 6

Supplementary Note 1: Power Spectra

Supplementary Figure 1 shows the power spectra generated using IBI tachograms from Fig. 4 for each maneuver. The location of spectral peaks corresponds to the respiratory rate expected from each breathing pattern. For example, for the inhale to exhale ratio of 4:4, 4:6, and 5:7 seconds in the resonant breathing exercises (left panel), the fundamental breathing frequency is 0.125, 0.1, and 0.083 Hz, indicating a respiratory rate of 7.5, 6, and 5 breaths per minute, respectively. The low-frequency (LF) and high-frequency (HF) bands marked on the power spectra are defined as the 0.04-0.15 Hz and 0.15-0.4 Hz regions, respectively. The integrated power in the LF and HF bands can distinguish controlled breathing from spontaneous breathing. They are also commonly used in the literature to assess the activity of the parasympathetic nervous system[1].

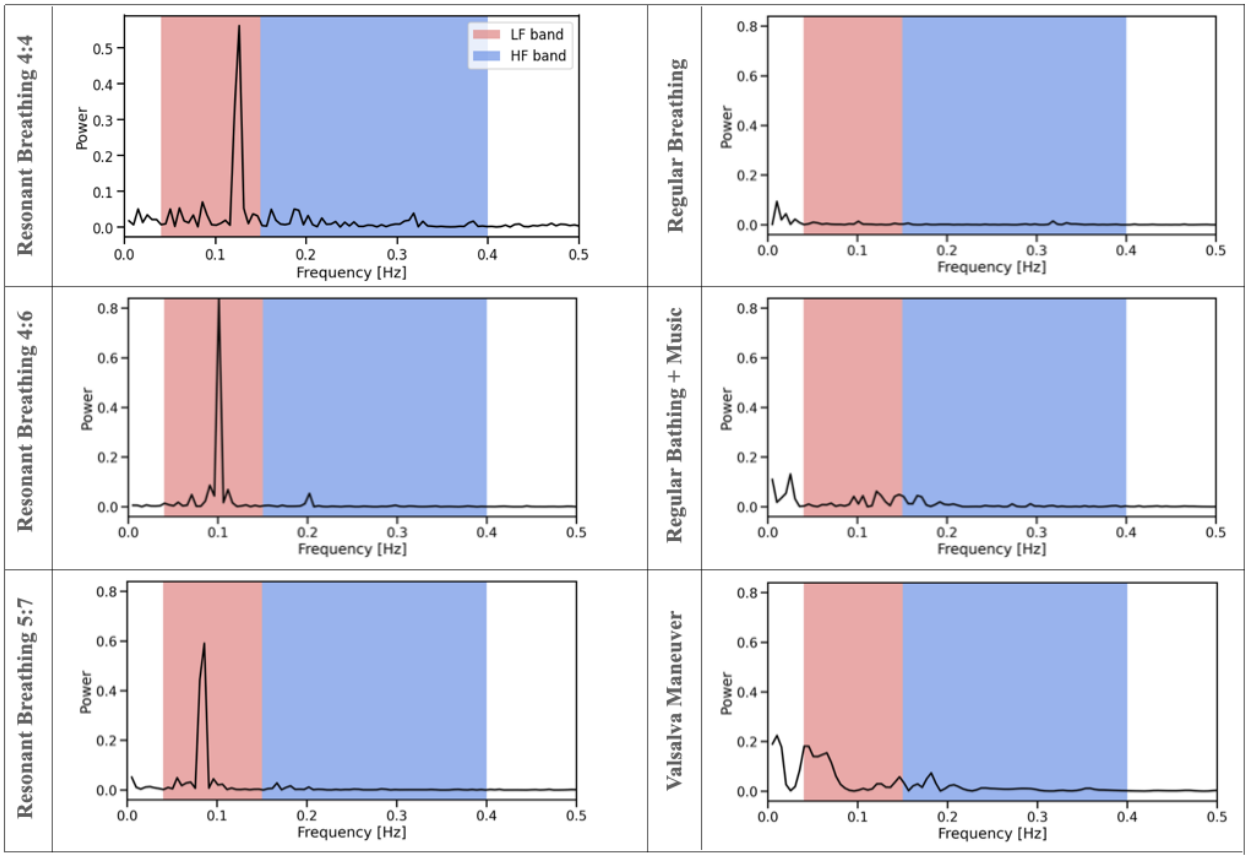

Supplementary Figure 1: Power spectra of interbeat-interval tachograms. Power spectra of interbeat-interval tachograms from Figure 4 for (left panel, from top to bottom) resonant breathing with the 4:4, 4:6, and 5:7 seconds of inhale to exhale ratio, and for (right panel, from top to bottom) regular breading, regular breathing while listening to music, and Valsalva maneuver; marked regions correspond to the low-frequency (LF) and high-frequency (HF) bands.

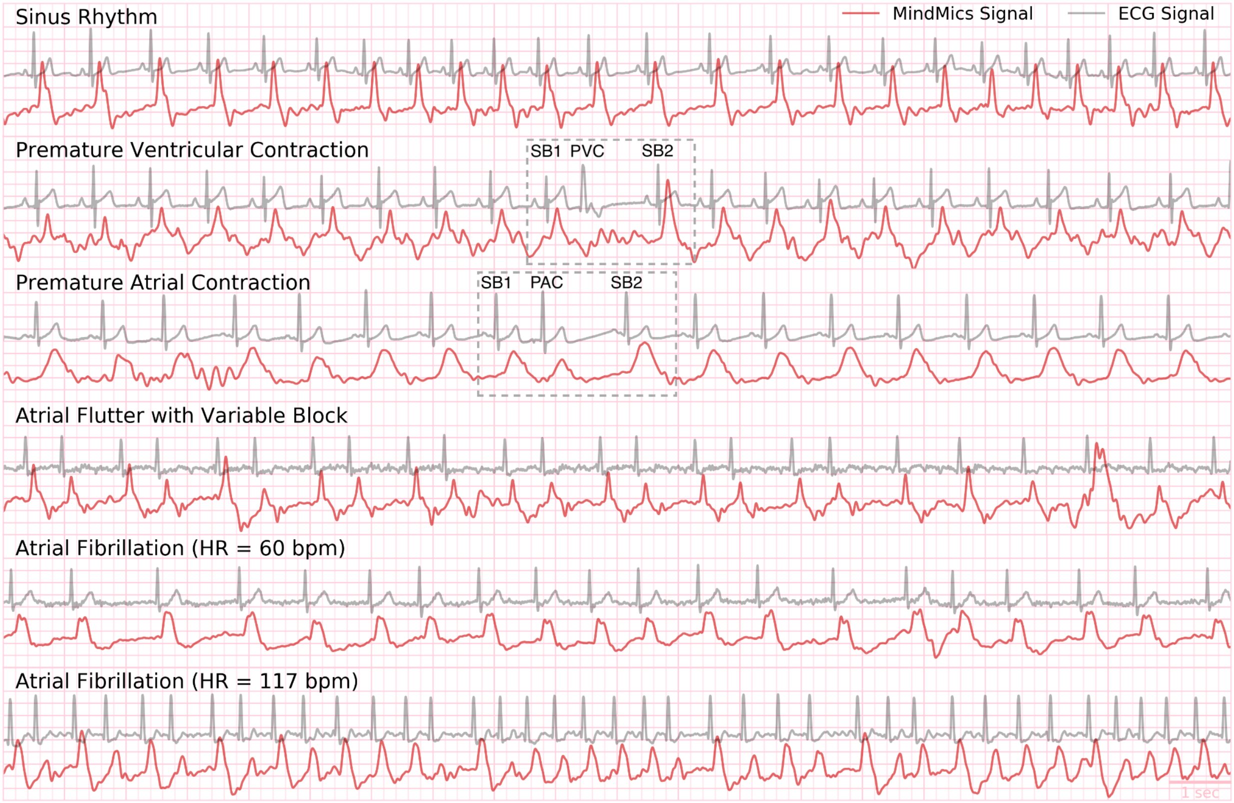

Supplementary Figure 2: **Cardiac rhythms observed in subjects from AF Study.** Examples of 20-second segments with different rhythms observed with IH (red) and ECG (gray). The segments illustrate the following (from top to bottom): Sinus Rhythm, Premature Ventricular Contraction, Premature Atrial Contraction, Atrial Flutter with Variable Block, Atrial Fibrillation with normal heart rate, Atrial Fibrillation with high heart rate. HR: heart rate, bpm: beats per minute, PVC: Premature Ventricular Contraction, PAC: Premature Atrial Contraction, SB1/SB2: sinus beat preceding/following PVC or PAC.

Supplementary Table 1: **Characteristics of recruited healthy SR patients.** Sex and age, rounded to the nearest 5 years, of SR patients. Information about the comorbidities was not collected during the study.

**Subject No.**	**Sex**	**Age (yr)**
MMIRB_SR_001	Female	30
MMIRB_SR_002	Male	35
MMIRB_SR_003	Male	25
MMIRB_SR_004	Male	35
MMIRB_SR_005	Male	35
MMIRB_SR_006	Male	35
MMIRB_SR_007	Male	65
MMIRB_SR_008	Male	40
MMIRB_SR_009	Male	55
MMIRB_SR_010	Male	60
MMIRB_SR_011	Male	75
MMIRB_SR_012	Female	25
MMIRB_SR_013	Male	35
MMIRB_SR_014	Female	35
MMIRB_SR_015	Male	60
MMIRB_SR_016	Male	25
MMIRB_SR_017	Male	60
MMIRB_SR_018	Female	20
MMIRB_SR_019	Female	20
MMIRB_SR_020	Female	55
MMIRB_SR_021	Female	50
MMIRB_SR_022	Female	40
MMIRB_SR_023	Male	25
MMIRB_SR_024	Female	60
MMIRB_SR_025	Male	55

Supplementary Table 2: **Characteristics of recruited AF patients.** Sex, age (rounded to the nearest 5 years), diagnosis, and comorbidities of AF patients. The dagger symbol marks two patients excluded from study, who were not in AF at the time of data collection.

**Subject No.**	**Sex**	**Age (yr)**	**Diagnosis**	**Comorbidities/Other medical conditions**
PRISMA_AF_001†	Female	80	Atrial fibrillation with atrial flutter	Hypertension, Chronic Obstructive Pulmonary Disease (COPD)
PRISMA_AF_002	Male	90	Atrial fibrillation	Mechanical aortic valve replacement, Coronary artery disease
PRISMA_AF_003	Female	80	Atrial fibrillation	
PRISMA_AF_004	Male	45	Atrial fibrillation with atrial flutter	Cardiomyopathy, hypertension, tricuspid valve regurgitation, hyperlipidemia, history of aortic aneurysm dissection
PRISMA_AF_005	Male	85	Atrial fibrillation	Hypertension, hyperlipidemia
PRISMA_AF_006	Male	75	Atrial fibrillation	Hypertension, history of ablation for atrial fibrillation
PRISMA_AF_007†	Male	90	Atrial fibrillation	
PRISMA_AF_008	Female	65	Permanent atrial fibrillation	
PRISMA_AF_009	Female	75	Atrial fibrillation	Thoracic aneurysms and COPD
PRISMA_AF_010	Male	70	Atrial fibrillation	
PRISMA_AF_011	Female	85	Atrial fibrillation	Aortic regurgitation, aortic stenosis, hypertension
PRISMA_AF_012	Male	70	Permanent atrial fibrillation	
PRISMA_AF_013	Female	85	Atrial fibrillation	Mitral valve regurgitation
PRISMA_AF_014	Female	65	Permanent atrial fibrillation	Hypertension, hyperlipidemia, COPD, Gastroesophageal reflux disease(GERD)
PRISMA_AF_015	Male	65	Atrial fibrillation	Cardiomyopathy, hypertension, hyperlipidemia
PRISMA_AF_016	Male	55	Atrial fibrillation	Hypertension, hyperlipidemia
PRISMA_AF_017	Female	60	Atrial fibrillation	

Supplementary References

1. Shaffer, F. & Ginsberg, J. P. An Overview of Heart Rate Variability Metrics and Norms. *Front. Public Health* **5**, 258 (2017).
