# Supplementary material for: In-ear infrasonic hemodynography with a digital health device for cardiovascular monitoring using the human audiome
Source: NPJ Digit Med. 2022 Dec 22;5:189. doi: 10.1038/s41746-022-00725-3 (PMC9780339; doi:10.1038/s41746-022-00725-3)
Supplement: Supplementary file 2 — written consent, human subject picture [file 41746_2022_725_MOESM2_ESM.pdf]

EMPLOYEE PHOTO RELEASE FORM

I, **John Cunsolo**, an employee of **MindMics, Inc.**, give my permission to its legal representatives and assigns, those for my employer is acting, and those acting with its permission, or its employees, the permission to take photographs of me and use them for any legal purpose.

I understand that I will not be paid for these photographs and have no rights to them. I am participating as a volunteer. I hereby waive any right to inspect or approve the finished photograph or advertising copy or printed matter that may be used in conjunction therewith or to the eventual use that it might be applied. I release my employer, its officers, employees and agents, from any and all claims of harm and liability as a result of any distortion, blurring, or alteration, optical illusion, or use in composite form, either intentionally or otherwise which may occur from making, showing, using or distributing these photographs/video.

I HAVE READ THIS RELEASE AND CONSENT FORM BEFORE AFFIXING MY SIGNATURE BELOW, AND I UNDERSTAND AND AGREE TO ITS TERMS.

John Cunsolo   November 15th, 2022

John Cunsolo   **Date**
