# Supplementary material for: In-ear infrasonic hemodynography with a digital health device for cardiovascular monitoring using the human audiome
Source: NPJ Digit Med. 2022 Dec 22;5:189. doi: 10.1038/s41746-022-00725-3 (PMC9780339; doi:10.1038/s41746-022-00725-3)
Supplement: Supplementary file 3 — Clinical Trial information - 1 [file 41746_2022_725_MOESM3_ESM.pdf]

In-ear infrasonic hemodynography with a digital health device for cardiovascular monitoring using the human audiome

F. R. Gilliam III *et al.*

CLINICAL TRIAL INFORMATION

Sinus Rhythm sample

The protocol was approved by the New England institutional review board (ClinicalTrials.gov Identifier: NCT05095753; start date: November 15, 2019, ongoing). Consecutive 25 healthy subjects (ages between 20 and 77 years, with the mean age of 42 years, and 35% female) were recruited at MindMics Inc. in Cambridge, MA for prospective study of clinical validation of the in-ear headphones with embedded IH technology with simultaneously captured ECG waveforms. All study subjects provided written informed consent. Study subjects wore the IH earbuds in their left and right ears and were fitted with different-sized eartips to ensure a proper occlusion. A medical-grade 3 lead ECG (GE Transport Pro) was connected to each study subject's left arm, right arm, and left leg to obtain reference signals. A time-series dataset was acquired through synchronized IH and ECG. Data for both IH and ECG devices were recorded with a sampling frequency of 1000 Hz. Data collection started with subjects seated upright and breathing normally. Subjects were then asked to perform a series of breathing maneuvers and introduced to soothing music to intentionally change respiratory rate and HR for a larger subset of data. The signals were collected using a data acquisition device (DAQ) and sent to a laptop computer over a wired USB connection. The computer encrypted the signal and securely sent it to the cloud infrastructure for storage and further processing.

Atrial Fibrillation Sample

The prospective study recruiting AF patients was approved by the institutional review board of The University of South Carolina School of Medicine (ClinicalTrials.gov Identifier: NCT05103579; start date: March 24, 2020, end date: January 31, 2021). The objective of this study was to evaluate the efficacy of the IH waveforms to monitor cardiac activity from participants with known atrial fibrillation. Consecutive study subjects were enrolled at Prisma Health in Sumter, SC and underwent simultaneous IH and ECG recording for 20 minutes. Each study subject provided written informed consent. Study subjects with a known history of AF (n=17) were screened and those in a rhythm of atrial fibrillation were included for participation (n=15, ages between 45 and 90 years, the mean age of 71 years, and 47% women). The clinical setup for the data collection process was identical to the one used for the Sinus Rhythm sample.
