# Supplementary material for: In-ear infrasonic hemodynography with a digital health device for cardiovascular monitoring using the human audiome
Source: NPJ Digit Med. 2022 Dec 22;5:189. doi: 10.1038/s41746-022-00725-3 (PMC9780339; doi:10.1038/s41746-022-00725-3)
Supplement: Supplementary file 4 — Clinical Trial information - 2 (SR) [file 41746_2022_725_MOESM4_ESM.pdf]

Title of Research - Data collection for Heart Rate and Blood Pressure
Sponsor Name - MindMics
Investigator Name - Dr. Anna Barnacka, CEO, MindMics
Site Address – 90 Sherman Street, Cambridge, MA, 02140
Contact information: 857-210-9181, anna.barnacka@mindmics.com

ii. Purpose of the study and background:

The purpose of the study is data collection for building and validating the algorithms for analytics of vital signs like Blood Pressure, Heart Rate, Stress, and Respiration Rate using wearable earphones (MindMics device).

Background: MindMics is developing technology to enhance an individual's quality of life by continuously monitoring the heart rate, stress, blood pressure thereby allowing patients to make better decisions regarding their health as they are experiencing symptoms and becoming aware of them. MindMics has a working prototype of the ear buds will perform all the conventional tasks earbuds do (listen to music, etc.), but, in addition, will measure your heart rate, stress, blood pressure. The earbuds capture the sounds made by the various cardiac structures pulsing and moving blood. The sound is caused by the acceleration and deceleration of blood and turbulence developed during rapid blood flow.

iii. Criteria for subject selection

The number of subjects to be enrolled is approximately 100-300.

Inclusion Criteria:

Ages 18-90, no gender restrictions. This range is selected because the vital signs and stress being recorded are subjective to all ages

Pregnant women can also be part of the study since there are no foreseen risks involved with the wearable earphones.

In the judgment of the investigator, has the cognitive ability to consent.

Exclusion criteria:

Patients with Pacemakers

The earbuds do not require a normal functioning ear. The earbuds capture the sounds made by the various cardiac structures pulsing and moving blood. The sound is caused by the acceleration and deceleration of blood and turbulence developed during rapid blood flow. Hence, even a deaf person would be considered.

Vulnerable subjects:

The device does not pose any risks to the vulnerable population. The device is non-invasive and simply involves wearing the earphones in a private room normally like any other earphones. The earphones do not alter or manipulate day-to-day activities of the subject or cause any harm to the subjects. The overall risk of the device in minimum.

iv. Methods and procedures:

After consent has been obtained,

1. Data collection will take place in a private room in the presence of 2-3 members of the MindMics team. Subjects will fill out a consent form prior to the experiment
2. After completing the consent form subjects are asked to sit in a lounge chair and the details of the experiment are conveyed to them, namely that the experiment consists of 6 major parts; a) the subjects will be asked to try different sets of earbuds until the right signal is received. b) Subject will be assisted with placing ECG leads to collect data c) Subjects will be asked to have their blood pressure taken. d) The subjects will then be asked to do a 20-30minute meditation/take deep breaths or listen to a ted talk to relax. e) After this period, the subjects will have their blood pressure taken again. f) The subjects will be debriefed about the observed data signal.
3. Once this is conveyed to the subject and they again have vocalized their consent the earbud fitting begins, subjects are first asked if they have a preference for earbud size, if so they are given their preferred size to try first, if not they are given a set of medium size. While trying different sets of earbuds, subjects are instructed to place the earbuds in their ears angled downwards towards their throat. In some cases, subjects are instructed to try and place the earbuds deeper in the ear canal but are instructed that this should never be to the point of being uncomfortable. If the first earbud size does not result in a good signal, the subjects may be given smaller or larger sizes and the fitting process is repeated until a good signal is achieved and the subject is comfortable.
4. Following the earbud fitting, the subject is asked to place three ECG leads according to the instructions provided with the ECG .
5. Next, subjects' blood pressure is taken via radial and brachial electronic sphygmomanometers (blood pressure cuff). These blood pressure measurements will be recorded as part of the data collection.
6. After the blood pressure measurement is taken the subjects are instructed to sit comfortably and the guided meditation session begins. The sessions take approximately 40 mins.
7. After the 20-30 minute meditation session is complete the subject's blood pressure is again measured using the same devices mentioned previously. This blood pressure measurement is also recorded as part of the data collection.
8. The subjects are then debriefed on the nature of the signal and what specific measurements were observed. The subjects are told the signal is a recording of their cardiovascular rhythms, and are shown the waveform of their heart. The waveform is explained in depth to the subject if they express interest.

9.

The whole process is expected to take 40 mins.

Data analysis and data monitoring:

The Heart Rate, Respiratory Rate, Heart Rate Variability will then be calculated from the waveform obtained in the experiment which will be used to validate hypothesis regarding the nature of the signal received from the left and the right eat and as training data to machine learning algorithms.

Data storage and confidentiality:

This calculated data will be retained in our internal database with no reference to the patient's name or any information. Access will be provided only to the MindMics team to analyze data for training algorithms to detect irregular heart rate, arrhythmia, blood pressure and other vital signs that might be found during analysis. MindMics will not be collecting sensitive personal identification information such as Social Security Number or Personal Health Identification information such as healthcare provider, health history record. Data will not be released to any agency or personnel. This is only for internal data analysis. However, the outcome of data analysis might be shared with clinicians to verify the hypothesis and the results can be used for publications.

. v. Risk/Benefit assessment:

There is no potential harm or injury associated with the research. The probability and magnitude of harm or discomfort anticipated in the research are not greater than those ordinarily encountered in daily life or during the performance of routine physical or psychological examinations or tests. There are no potential risks with respect to psychological, sociological, economic, or legal. The overall risk of the device in minimum.

Potential benefits to the subjects:

There are no direct benefits to the subjects

Alternatives to participation: This is not applicable since the device is not treating or diagnosing any critical medical conditions.

Method of subject identification and recruitment:

The subjects will be identified and recruited via word of mouth or advertising on social media networks like Facebook and Instagram. The subjects interested can either walk-in or call prior to their arrival. The subjects will be recruited and identified by a unique subject number. Personal identification information will not be stored.

Process of consent:

Data Analyst or the PI are authorized to obtain consent. The subject will be explained the details of the consent form. The subject is later allowed to read the consent form as per their convenience in a private room. The subject's participation or withdrawal is voluntary. The consent form will then be stored in paper format after assigning subject number to the participating subject.

Subject capacity: If the subject does not speak or read English, a translator in the appropriate language will be involved. If any subject is differently abled, appropriate arrangements will be made

to accommodate the subjects' needs prior to their arrival. Since the study is minimal risk and does not involve treatment, diagnosis or prevention of any disease, there are no anticipated risks associated with the capacity of any subject.

Subject/representative comprehension:

The subject will be explained the details of the consent form. The subject is later allowed to read the consent form as per their convenience in a private room. The subject's participation or withdrawal is voluntary. The PI or the Data Analyst will also highlight important information from consent form to help guide discussion.

Costs to the subject: There are no significant costs involved in conducting this study or follow-up. The subjects may incur travel costs. The subjects will not be charged for their participation in the study.

Payment for participation: The subjects will not be paid for their participation in the study.
