# Supplementary material for: In-ear infrasonic hemodynography with a digital health device for cardiovascular monitoring using the human audiome
Source: NPJ Digit Med. 2022 Dec 22;5:189. doi: 10.1038/s41746-022-00725-3 (PMC9780339; doi:10.1038/s41746-022-00725-3)
Supplement: Supplementary file 5 — Clinical Trial Information - 3 (AF) [file 41746_2022_725_MOESM5_ESM.pdf]

Design and rationale of the assessment of the Mindmics earbuds recording system to collect data to facilitate the development of an algorithm to discriminate atrial fibrillation from sinus rhythm

Investigator:

F. Roosevelt Gilliam III, MD, FACP,FACC,FHRS
Palmetto Health USC Medical Group
Rosey.gilliam@prismahealth.org

Short Title: MindMics AF

Background

MindMics earbuds is a new technology designed enhance an individual's quality of life by continuously monitoring the heart rate, stress, and blood pressure allowing patients to make decisions regarding their health. MindMics has a working prototype of the ear buds which will perform all the conventional tasks of earbuds (listen to music, etc.), but, in addition, will measure heart rate, stress, blood pressure. The earbuds capture the sounds made by the various cardiac structures pulsing and moving blood. The sound is caused by the acceleration and deceleration of blood and the turbulence developed during rapid blood flow.

Abstract

Atrial fibrillation is present in over 5,000,000 Americans and has been associated with increased risks for stroke, debilitation, hospitalization and early death. Identified patients (with atrial fibrillation) are treated with anticoagulation agents reducing the occurrence of these events. Many patients do not recognize they have atrial fibrillation until suffering a catastrophic stroke. There has been an effort to use insertable loop recorders (ILRs) to record heart rhythms of those patients felt likely to have had asymptomatic atrial fibrillation. This emphasis focuses particularly on those patients suffering a stroke without obvious etiology (cryptogenic stroke). MindMics offers a new technology, using earbuds to evaluate the cardiac rhythm noninvasively, with a potential to identify atrial fibrillation in patients who may be entirely asymptomatic.

ECG recording devices attached to Smart phones enable laypersons to record a high fidelity electrocardiogram, in many instances detect, and diagnose clinical arrhythmias prior to exhibiting outward symptoms. These devices are extraordinarily accurate in recording electrocardiogram data are limited as they are only effective for very short intervals typically 30 seconds while the patient actively records the cardiac rhythm. The MindMics recording device utilizes earbuds that can play music as well as record auditory signals, which can evaluate a number of clinical targets as well as the patient's cardiac rhythm. This study will collect information that will be used to develop algorithms with the ability to discern differences between atrial fibrillation and normal sinus rhythm.

Patients enrolled in the study will use the MindMics earbuds device to record atrial fibrillation and simultaneously ECG recordings using the typical electrocardiogram recordings. These recordings will be used to develop algorithms that will enable the Mindmics device to discern normal sinus rhythm from atrial fibrillation.

MindMics Background

MindMics has developed technology to enhance an individual's quality of life by continuously monitoring the heart rate, stress, blood pressure allowing patients to make better decisions regarding their health as they become aware of symptoms. MindMics has a working prototype of the ear buds which perform all the conventional tasks earbuds do (listen to music, etc.), but will measure heart rate, stress, and blood pressure. The earbuds capture the sounds made by the various cardiac structures pulsing and moving

blood. Acceleration and deceleration of blood causes the sound and the turbulence developed during rapid blood flow.

Introduction:

This study will evaluate the efficacy of the MindMics earbud recording apparatus to record data from patients with known atrial fibrillation as well as those in sinus rhythm. Patients in the clinic and patients with atrial fibrillation scheduled to undergo elective direct-current cardioversion will be the chosen subjects. This process will select a variety of patients to allow evaluation of the Mindmics apparatus's ability to collect data from patients with normal as well as abnormal cardiac rhythms.

Hypotheses

The MindMics earbud device is capable of recording normal cardiac rhythms in patients noninvasively using earbud device in a noninvasive manner. The same apparatus may be capable of discerning atrial fibrillation compared with normal sinus rhythm. This will be evaluated by collecting information from patients with atrial fibrillation in the office as well as those scheduled to undergo an elective direct-current cardioversion procedure. A timed recording of the clinical rhythm while wearing the apparatus will allow simultaneous recording of data from an ECG recording (documenting the patient's clinical arrhythmia) at the same time the MindMics earbud records its data obtained during the ECG recording of the patient's rhythm. For those patient scheduled for direct current cardioversion, the patient will undergo the direct-current cardioversion and a second recording will be obtained during sinus rhythm afterwards allowing an appropriate comparison of the differences in the recording sets.

The objective is to demonstrate differences between recordings made in atrial fibrillation and those in sinus rhythm that can be used to develop an algorithm allow discernment between atrial fibrillation and sinus rhythm.

OVERALL DESIGN

This study is designed to collect sequential patient's in a nonrandomized unblinded procedure designed to collect information using the apparatus to document its capability of recording cardiac rhythm data, then using this recorded data to determine whether the patient has an abnormal cardiac rhythm. Initial efforts will be directed to develop an algorithm to detect atrial fibrillation.

Patients will be recruited from inpatients at Palmetto Tuomey Medical center or in the outpatient office during routine office visits for patients with clinical arrhythmias for elective recording for group 1. Group 2 patients will be recruited prior to direct-current cardioversion. Both groups will

be explained the study, what is their participation, potential risk and expectations for their participation in the study.

Once selected each patient will be required to sign a study protocol consent form before actual entry into the study. Once the consent is completed, the patients will be connected to the Mindmics apparatus as well as an electrocardiographic recording system. The study participant will lie quietly collecting simultaneous electrocardiographic data and data from the Mindmics earbuds. Group 1 patients will be identified as outpatients during their routine office visits will have 15 minutes of recording in a patient examination room during the office visit. Group 2 patients undergoing Direct-Current Cardioversion will have 15 minutes of recording prior to the cardioversion procedure and 15 minutes of recording after completion of the cardioversion procedure.

NUMBER OF PARTICIPANTS

Approximately 25 patients will be recruited for group 1 from inpatient or outpatient office visits. These patients will have persistent atrial fibrillation during the office visit. If necessary would consider increasing the total number of patients to 50 in this group

Approximately 25 patients will be recruited for group 2 prior to direct-current cardioversion allowing collection of data from before and after cardioversion to document differences in recordings during sinus rhythm after having successful cardioversion from atrial fibrillation. While certainly some patients will revert back to atrial fibrillation and not have periods of sinus rhythm is anticipated the vast majority of patients will have successful cardioversions long enough to record at least the 15 minutes post cardioversion in sinus rhythm. If necessary would consider increasing the total number of patients to 50 in this group

DURATION

The total duration of involvement for each study participant is for the duration of the recording interval. Group 1 participants will have a 15-minute recording interval while those in group to will have 2 sessions of 15 minutes each separated by the direct current cardioversion. No further participation will be required.

All data collected during the study will be kept by Mindmics. All data collected in the study will be de-identified

OUTCOME MEASURES and STATISTICAL ANALYSIS

Each recording will be evaluated by Mindmics to evaluate the ability of the recording apparatus to provide data that would lead to clear discernment of the patient's cardiac rhythm as well as rate related

phenomena. At this juncture, it is anticipated the collected data will be used to develop and test algorithms yet to be developed and to make clinical determinations of the cardiac rhythms.

Initial data will be evaluated to determine if the Mindmics earbuds are capable of discriminating atrial fibrillation from normal sinus rhythm. For that reason, it is not possible to suggest a statistical method until it is determined the device data can be used to evaluate differences in cardiac rhythm.

SAFETY

There is no potential harm or injury associated with the research. The probability and magnitude of harm or discomfort anticipated in the research are not greater than those ordinarily encountered in daily life or during the performance of routine physical or psychological examinations or tests. There are no potential risks with respect to psychological, sociological, economic, or legal. The overall risk of the device in minimum.

Potential Benefit to Participants

This study is performed in such a manner that it is not anticipated there would be an ability to provide diagnostic data that may be helpful for the study participants.

References

McConnell MV, Turakhia MP, Harrington RA, King AC, Ashley EA. *Mobile Health Advances in Physical Activity, Fitness, and Atrial Fibrillation: Moving Hearts.* J Am Coll Cardiol. 2018 Jun 12;71(23):2691-2701. doi: 10.1016/j.jacc.2018.04.030.

Ahmed M. Al-Kaisey, Anoop N. Koshy, Francis J. Ha, Ryan Spencer, Liam Toner, Jithin K. Sajeev, Andrew W. Teh, Omar Farouque and Han S. Lim. *Accuracy of wrist-worn heart rate monitors for rate control assessment in atrial fibrillation.* International Journal of Cardiology, 10.1016/j.ijcard.2019.11.120, (2019).

Sajeev JK, Koshy AN, Teh AW. *Wearable devices for cardiac arrhythmia detection: a new contender?* Intern Med J. 2019 May;49(5):570-573. doi: 10.1111/imj.14274.

Mark DB, Anstrom KJ, Sheng S, Piccini JP, Baloch KN, Monahan KH, Daniels MR, Bahnson TD, Poole JE, Rosenberg Y, Lee KL, Packer DL; CABANA Investigators. *Effect of Catheter Ablation vs Medical Therapy on Quality of Life Among Patients With Atrial Fibrillation: The CABANA Randomized Clinical Trial*. JAMA. 2019 Apr 2;321(13):1275-1285. doi: 10.1001/jama.2019.0692. Erratum in: JAMA. 2019 Jun 18;321(23):2370.

Packer DL, Mark DB, Robb RA, Monahan KH, Bahnson TD, Moretz K, Poole JE, Mascette A, Rosenberg Y, Jeffries N, Al-Khalidi HR, Lee KL; CABANA Investigators. *Catheter Ablation versus Antiarrhythmic Drug Therapy for Atrial Fibrillation (CABANA) Trial: Study Rationale and Design.* Am Heart J. 2018 May;199:192-199. doi: 10.1016/j.ahj.2018.02.015. Epub 2018 Mar 7.
