# Supplementary material for: In-ear infrasonic hemodynography with a digital health device for cardiovascular monitoring using the human audiome
Source: NPJ Digit Med. 2022 Dec 22;5:189. doi: 10.1038/s41746-022-00725-3 (PMC9780339; doi:10.1038/s41746-022-00725-3)
Supplement: Supplementary file 6 — Reporting Summary checklist [file 41746_2022_725_MOESM6_ESM.pdf]

nature portfolio

Corresponding author(s): Anna Barnacka

Last updated by author(s): Sep 26, 2022

Reporting Summary

Nature Portfolio wishes to improve the reproducibility of the work that we publish. This form provides structure for consistency and transparency in reporting. For further information on Nature Portfolio policies, see our Editorial Policies and the Editorial Policy Checklist.

Statistics

For all statistical analyses, confirm that the following items are present in the figure legend, table legend, main text, or Methods section.

n/a	Confirmed	
☐	☒	The exact sample size (*n*) for each experimental group/condition, given as a discrete number and unit of measurement
☐	☒	A statement on whether measurements were taken from distinct samples or whether the same sample was measured repeatedly
☐	☒	The statistical test(s) used AND whether they are one- or two-sided<br>*Only common tests should be described solely by name; describe more complex techniques in the Methods section.*
☒	☐	A description of all covariates tested
☐	☒	A description of any assumptions or corrections, such as tests of normality and adjustment for multiple comparisons
☐	☒	A full description of the statistical parameters including central tendency (e.g. means) or other basic estimates (e.g. regression coefficient) AND variation (e.g. standard deviation) or associated estimates of uncertainty (e.g. confidence intervals)
☐	☒	For null hypothesis testing, the test statistic (e.g. *F*, *t*, *r*) with confidence intervals, effect sizes, degrees of freedom and *P* value noted<br>*Give P values as exact values whenever suitable.*
☒	☐	For Bayesian analysis, information on the choice of priors and Markov chain Monte Carlo settings
☒	☐	For hierarchical and complex designs, identification of the appropriate level for tests and full reporting of outcomes
☐	☒	Estimates of effect sizes (e.g. Cohen's *d*, Pearson's *r*), indicating how they were calculated

Our web collection on statistics for biologists contains articles on many of the points above.

Software and code

Policy information about availability of computer code

Data collection	Data was collected simultaneously from earbuds and ECG using customary code written in python version 3.7.3. Data was subsequently sent and stored in a proprietary cloud repository using version 1.8 of the InfluxDB software.
Data analysis	Data analysis was conducted using customary code written in python 3.7.12 with python scientific libraries including numpy v1.21.5, pandas v1.3.4, scipy v1.5.4, scikit-learn v0.22.2, and matplotlib v.3.3.4.

For manuscripts utilizing custom algorithms or software that are central to the research but not yet described in published literature, software must be made available to editors and reviewers. We strongly encourage code deposition in a community repository (e.g. GitHub). See the Nature Portfolio guidelines for submitting code & software for further information.

Data

Policy information about availability of data

All manuscripts must include a data availability statement. This statement should provide the following information, where applicable:

- Accession codes, unique identifiers, or web links for publicly available datasets
- A description of any restrictions on data availability
- For clinical datasets or third party data, please ensure that the statement adheres to our policy

Datasets used for the analyses in this study are available from the corresponding author upon request.

Field-specific reporting

Please select the one below that is the best fit for your research. If you are not sure, read the appropriate sections before making your selection.

☒ Life sciences ☐ Behavioural & social sciences ☐ Ecological, evolutionary & environmental sciences

For a reference copy of the document with all sections, see nature.com/documents/nr-reporting-summary-flat.pdf

Life sciences study design

All studies must disclose on these points even when the disclosure is negative.

Sample size	Datasets comprise 25 study subjects in sinus rhythm and 17 study subjects in AF rhythm.
Data exclusions	Two study subjects were excluded from the AF sample for anaysis, as they were not in the AF rhythm at the time of data collection.
Replication	Experimental findings are easily replicable. Datasets used in the analysis are available from the corresponding author upon request. Some models and code generated or used during the study are proprietary or confidential in nature and may only be provided with restrictions.
Randomization	No randomization was performed during patient recruitment. Participants were recruited without any age, gender, or racial bias.
Blinding	Anonymous IDs were assigned to all study subjects to protect their privacy. No blinding was conducted, as it does not impact the performance comparison between earbuds and gold-standard ECG.

Reporting for specific materials, systems and methods

We require information from authors about some types of materials, experimental systems and methods used in many studies. Here, indicate whether each material, system or method listed is relevant to your study. If you are not sure if a list item applies to your research, read the appropriate section before selecting a response.

Materials & experimental systems

n/a	Involved in the study	
☒	☐	Antibodies
☒	☐	Eukaryotic cell lines
☒	☐	Palaeontology and archaeology
☒	☐	Animals and other organisms
☐	☒	Human research participants
☐	☒	Clinical data
☒	☐	Dual use research of concern

Methods

n/a	Involved in the study	
☒	☐	ChIP-seq
☒	☐	Flow cytometry
☒	☐	MRI-based neuroimaging

Human research participants

Policy information about studies involving human research participants

Population characteristics	In the SR study, 25 healthy subjects were of ages between 20 and 77 years, with the mean age of 42 years, and 35% were female. In the AF study, 15 subjects with known history of AF and in AF confirmed at the time of data collection were of ages between 45 and 90 years, with the mean age of 71 years, and 47% were female.
Recruitment	The SR patients were volunteers who agreed to have their data taken at an in-office setting and who had no history of cardiovascular diseases. Study subjects for AF were patients in a general cardiology practice as outpatients or hospitalized patients, required to have a know history of AF. The minimum age of participants in both groups was required to be 18 years.
Ethics oversight	The SR study protocol was approved by the New England institutional review board. The study recruiting AF patients was approved by the institutional review board of The University of South Carolina School of Medicine. Participants provided written informed consent to participate in the studies.

Note that full information on the approval of the study protocol must also be provided in the manuscript.

Clinical data

Policy information about clinical studies

All manuscripts should comply with the ICMJE guidelines for publication of clinical research and a completed CONSORT checklist must be included with all submissions.

Clinical trial registration	ClinicalTrials.gov Identifier: NCT05095753 for the SR study and ClinicalTrials.gov Identifier: NCT05103579 for the AF study.

Study protocol	Trial details can be accessed at https://clinicaltrials.gov/ct2/show/NCT05095753 and https://clinicaltrials.gov/ct2/show/NCT05103579 for the SR and AF study, respectively. The study protocols are appended to the manuscript.
Data collection	For both studies, data from earbuds and ECG were collected simultaneously and sent to a cloud infrastructure for later analysis and storage, while subjects were seated upright and breathing normally. For SR study, subjects were additionally asked to perform breathing exercises of predefined duration. For the SR study, data collection started on November 15, 2019 and is ongoing. For the AF study, the data collection took place from March 24, 2020 to January 31, 2021.
Outcomes	The primary variables of the study were inter-beat intervals, heart rate and heart rate variability measured independently with earbuds and ECG. The comparison between earbud and ECG performance was investigated separately for study subjects in SR and AF rhythm.
